# Impact of Insertion Sequences and Recombination on the Population Structure of *Staphylococcus haemolyticus*

**DOI:** 10.1371/journal.pone.0156653

**Published:** 2016-06-01

**Authors:** Ons Bouchami, Herminia de Lencastre, Maria Miragaia

**Affiliations:** 1 Laboratory of Molecular Genetics, Instituto de Tecnologia Química e Biológica (ITQB) António Xavier, Oeiras, Portugal; 2 Laboratory of Bacterial Evolution and Molecular Epidemiology, Instituto de Tecnologia Química e Biológica (ITQB) António Xavier, Oeiras, Portugal; 3 Laboratory of Microbiology and Infectious Diseases, The Rockefeller University, New York, New York, United States of America; University Medical Center Utrecht, NETHERLANDS

## Abstract

*Staphylococcus haemolyticus* is one of the most common pathogens associated with medical-device related infections, but its molecular epidemiology is poorly explored. In the current study, we aimed to better understand the genetic mechanisms contributing to *S*. *haemolyticus* diversity in the hospital environment and their impact on the population structure and clinical relevant phenotypic traits. The analysis of a representative *S*. *haemolyticus* collection by multilocus sequence typing (MLST) has identified a single highly prevalent and diverse genetic lineage of nosocomial *S*. *haemolyticus* clonal complex (CC) 29 accounting for 91% of the collection of isolates disseminated worldwide. The examination of the sequence changes at MLST loci during clonal diversification showed that recombination had a higher impact than mutation in shaping the *S*. *haemolyticus* population. Also, we ascertained that another mechanism contributing significantly to clonal diversification and adaptation was mediated by insertion sequence (IS) elements. We found that all nosocomial *S*. *haemolyticus*, belonging to different STs, were rich in IS*1272* copies, as determined by Southern hybridization of macrorestriction patterns. In particular, we observed that the chromosome of a *S*. *haemolyticus* strain within CC29 was highly unstable during serial growth *in vitro* which paralleled with IS*1272* transposition events and changes in clinically relevant phenotypic traits namely, mannitol fermentation, susceptibility to beta-lactams, biofilm formation and hemolysis. Our results suggest that recombination and IS transposition might be a strategy of adaptation, evolution and pathogenicity of the major *S*. *haemolyticus* prevalent lineage in the hospital environment.

## Introduction

Among the coagulase-negative staphylococci (CoNS), *Staphylococcus haemolyticus* is one of the most important nosocomial human pathogens worldwide being mainly associated with bloodstream and device-associated infections [1–4]. Moreover, its relevance is expected to rise since implanted medical devices are increasingly used in clinical practice. In spite of its clinical relevance, its epidemiology, pathogenicity and evolutionary processes are not well understood.

Nosocomial isolates of *S*. *haemolyticus* are distinguished by their multidrug resistant pattern, high levels of resistance [5], formation of thick biofilms [3] and the frequent phenotypic variation [4], but the mechanisms associated with such characteristics are poorly explored. In contrast to *S*. *epidermidis*, the molecular basis of *S*. *haemolyticus* pathogenicity is elusive, but is probably related to putative hemolysins, adhesins, exonucleases, proteases, and genes encoding a capsular polysaccharide found in its genome [4,6]. Another virulence factor that is crucial for *S*. *haemolyticus* infectious process is its ability to produce biofilm, but the biofilm composition is largely unknown and the molecular mechanisms involved have not been elucidated. Unlike *S*. *aureus* and *S*. *epidermidis*, *S*. *haemolyticus* biofilms appear to be polysacharide intercellular adhesion (PIA)-independent and not associated with accumulation-associated protein (*Aap*) and biofilm associated protein (*Bhp*) [3].

Methicillin resistant *S*. *haemolyticus* (MRSHae) differs genetically from methicillin-susceptible *S*. *haemolyticus* (MSSHae) isolates by the presence, in the chromosome, of a large piece of foreign DNA, called the staphylococcal chromosome cassette *mec* (SCC*mec*), which carries the central element of methicillin resistance—the *mecA* gene [7]. The *mecA* gene encodes for an extra penicillin-binding protein, PBP2A, a transpeptidase with a low affinity for β-lactams [8]. SCC*mec* is a genomic island ranging in size from 21Kb to 67Kb which is inserted at the 3’ end of the *orfX* gene and located near the origin of replication. The SCC*mec* element is characterized by the presence of two essential loci: the *mec* gene complex, containing *mecA* and its regulators and the *ccr* gene complex, encoding recombinases, which are responsible for SCC*mec* mobility. Twelve different structural types of SCC*mec* (I to XII) have been identified so far in *S*. *aureus* [9,10] based on the combination of a *mec* complex class and a *ccr* allotype (http://www.sccmec.org/Pages/SCC_TypesEN.html). Much less is known about the genetics of methicillin resistance in CoNS.

By pulsed-field gel electrophoresis (PFGE) the few studies available showed that nosocomial *S*. *haemolyticus* are genetically diverse; however, the analysis of the population structure by MLST revealed a relative conservation of the core genome [11]. This observation was later confirmed by whole genome sequencing (WGS) that showed that a clonal expansion of nosocomial multiresistant *S*. *haemolyticus* isolates has occurred [12]. On the other hand, the typing of the SCC*mec* element in *S*. *haemolyticus* showed that SCC*mec* type 5C was the most common, but variants with deletions of *mecA* and the cassette chromosome *ccr* were also observed [4,13].

One of the most striking features of *S*. *haemolyticus* is the existence in its genome of as many as 82 insertion sequence (IS) elements [4], which is a number much higher than that found in all other species of staphylococci. IS are transposable elements (less than 2.5 kb) that carry no genetic information except for transposases and flanking short terminal inverted repeats which serve as recognition sites for the transposase. This specific enzyme normally excises the IS from one genetic location and inserts it into another (conservative transposition), but sometimes the IS replicates and a copy is inserted at a new chromosomal site (replicative transposition) [14,15]. The impact of IS in the overall genome architecture and gene expression can be very large, especially when present in multiple copies. These elements often cause gene inactivation and have strong polar effects, but can also lead to the activation or change of the expression of adjacent genes [13]. Moreover, they can lead to complex chromosomal rearrangements that result in inversions or deletions, with impact on host adaptation [16]. In spite of the very high number of IS in *S*. *haemolyticus*, the impact of these elements in genome architecture, population structure, and pathogenicity of *S*. *haemolyticus* was only explored for a very limited number of strains and never analyzed at the population level.

In order to better understand the molecular epidemiology and the evolutionary history of *S*. *haemolyticus* in the hospital environment as well as the impact of IS elements on its population structure and clinically relevant phenotypic traits, we analyzed a geographically diverse collection of nosocomial *S*. *haemolyticus* by a combination of genotypic and phenotypic tests.

## Materials and Methods

### Ethical statement

Individual ethical approval and informed consent was not required for this study since all *S*. *haemolyticus* isolates were obtained as part of routine clinical diagnostic testing and were analyzed anonymously. Moreover, in this study we analyzed the bacterial isolates, not the human subjects. The collection of samples was in accordance with the European Parliament and Council decision for the epidemiological surveillance and control of communicable disease in the European community (http://www.ecdc.europa.eu/en/activities/surveillance/EARS-Net/Pages/index.aspx and http://www.ecdc.europa.eu/en/activities/surveillance/EARS-Net/publications/Pages/documents.aspx).

### Bacterial isolates

A total of 133 geographically diverse *S*. *haemolyticus* nosocomial isolates (127 MRSHae and 6 MSSHae) were collected in 16 different countries in 1998 and 2010 from disease (n = 66 isolates) and from carriage (n = 26). No information on the remaining 41 isolates concerning disease or colonization origin was available. The representative collection included in this study comprised isolates originating from Argentina (n = 2 isolates), Brazil (n = 1), Bulgaria (n = 6), China (n = 19), Chile (n = 4), Colombia (n = 1), Denmark (n = 11), Hungary (n = 5), Iceland (n = 14), Italy (n = 19), Japan (n = 1), Mexico (n = 7), Poland (n = 11), Portugal (n = 28), Taiwan (n = 2) and Uruguay (n = 2). Clinical and demographic data available were recorded.

### Species identification

*S*. *haemolyticus* strains were identified by internal transcribed spacer PCR (ITS-PCR) as previously described [17].

### Antibiotic susceptibility testing

Antibiotic susceptibility testing by the disk diffusion test (Kirby-Bauer) was performed according to the guidelines of the Clinical and Laboratory Standards Institute (CLSI, 2014) [18]. A total of 14 antibiotics (purchased from Oxoid, Basingstoke, UK) were tested: oxacillin (OXA, 1 μg), cefoxitin (CFX, 30 μg), penicillin (PEN, 10 UI), gentamicin (GEN, 10 μg), ciprofloxacin (CIP, 5 μg), erythromycin (ERY, 15 μg), clindamycin (CLN, 2 μg), chloramphenicol (CHL, 30 μg), rifampin (RIF, 5 μg), tetracycline (TET, 30 μg), vancomycin (VAN, 30 μg), Teicoplanin (TE, 30 μg), trimethoprim-sulfamethoxazole (SXT, 1.25/23.75 μg) and quinupristin/dalfopristin (QDA, 15 μg).

Susceptibility to oxacillin (1 μg), cefoxitin (30 μg) and vancomycin (30 μg) were evaluated by the E-test (AB BioMérieux, Solna, Sweden) according to CLSI recommendations [18] on Mueller-Hinton agar (Becton, Dickinson and Company, Le Pont de Claix, France).

Isolates that presented resistance to three or more classes of antibiotics, other than β-lactams, were classified as having a multidrug resistance profile.

### DNA preparation

Agarose disks for pulsed-field gel electrophoresis (PFGE) were prepared as previously described for *S*. *aureus* [19]. Genomic DNA of pure cultures for PCR was extracted by the guanidine isothiocyanate extraction method as described by Couto *et al*. [17]. A DNA probe for the IS*1272* was prepared by PCR amplification with the specific oligonucleotides IS*1272*-1 (5’-TTCTAAGACAGATCATGATGCG-3’) and IS1*272*-2 (5’-TCCTCGGACAGACATCCG-3’), which were designed based on the sequence of *S*. *haemolyticus* JCSC1435 (GenbBank accession number AP00671, sequence position 792–1494). PCR was performed in 50 μl mixture containing 1X *Taq* DNA polymerase Buffer with 1.5 mM MgCl_2_ (Applied Biosystems, Foster City, CA, USA), 0.5 μM of each primer, 40 μM each deoxynucleoside triphosphate (MBI Fermentas, Hanover, MD), 1.25 U DNA *Taq* polymerase (Applied Biosystems, Foster City, CA, USA) and approximately 100ng (5 μL) of template DNA, and the mixture was subjected to a denaturation step of 4 min at 94°C; 30 cycles of 30s at 94°C, 1 min of annealing at 50°C, and 2 min of extension at 72°C; and a final elongation step of 2 min at 72°C in a T1 thermocycler (Biometra, Göttingen, Germany). PCR products were visualized after migration in 1% Seakem LE (Cambrex, Rock land, ME) agarose 1X Tris-acetate-EDTA (TAE, (Bio Rad, Hercules, CA) gel. The amplified product (700 bp) was purified by the Wizard PCR Preps DNA Purification System (Promega, Madison, WI, USA). The probe for *mecA* was amplified by PCR using previously described primers [20, 21] and purified. The following control strains were used as a source for the preparation of probes: *S*. *aureus* N315 (*mecA*) and *S*. *haemolyticus* JCSC1435 (IS*1272*).

### Detection of the *mecA*

The presence of the *mecA* gene was detected by amplification by PCR in all isolates as previously described [20] and confirmed for all the variants obtained in the stability assay by hybridizing the SmaI restriction band patterns with a DNA probe for *mecA*.

### PFGE analysis

The genetic relatedness of all *S*. *haemolyticus* isolates was determined by PFGE after digestion of the total DNA with SmaI according to Chung *et al*. [19]. The DNA banding patterns were analysed using BioNumerics^®^ software version v6.6 (Applied Maths, Saint-Martens-Latem, Belgium) with optimization set at 1.3% and position tolerance set at 0.8% [22]. The Dice coefficient of similarity was calculated, and the unweighted pair group method with arithmetic averages (UPGMA) was used for cluster analysis. As previously described [22], a cut-off at 78% similarity of the Dice coefficient was used to define a PFGE type. PFGE subtypes were defined by groups with different profiles that had a Dice coefficient higher than 78%. PFGE types were identified by letters; and subtypes were identified by letters followed by a numeric subscript.

### Southern blotting and DNA hybridization

SmaI DNA fragments in PFGE gels, were transferred by vacuum blotting as previously described [23] and hybridized with a DNA probe specific for IS*1272* using ECL direct Prime Labeling and detection systems (Amersham Biosciences, Buckinghamshire, United Kingdom), according to manufacturer’s instructions. The number of IS was estimated for each isolate by counting the number of IS*1272*-hybridizing bands appearing on the x-ray film. *S*. *aureus* COL strain was used as a positive control.

### Analysis of SCC*mec* structure

The structures of *ccr* and *mec* complexes were determined as described by Okuma *et al*. [21]. The combination of the type of *ccr* gene complex and the class of *mec* gene complex defined the SCC*mec* types [24,25]. SCC*mec* types were classified using the guidelines proposed by the International Working Group on the classification of Staphylococcal Cassette Chromosome Elements (IWG-SCC) [26].

### Estimate of SCC*mec* acquisition

Considering the evolutionary relationships as defined by eBURST as well as SCC*mec* typing results, we attempted to estimate the number of times SCC*mec* was acquired by *S*. *haemolyticus* as previously described [27,28].

### MLST analysis

MLST was performed on selected isolates using the new MLST scheme for *S*. *haemolyticus* described by Cavanagh *et al*. [11]. The isolates were selected based on PFGE and SCC*mec* typing data, representing one isolate from each PFGE subtype-SCC*mec* combination. The nucleotide sequences obtained were analyzed by using the software SeqMan (v5.03, DNASTAR, Inc.) and allele numbers were assigned following comparison of the DNA sequence with the sequences of MLST alleles provided by Cavanagh *et al*. [11]. For each isolate, the allele numbers at each of the seven loci defined the allelic profile or sequence type (ST). eBURST approach was used for cluster analysis of MLST data and clonal complexes (CC) were defined as previously described [29] using eBURST software version 3 (http://eburst.mlst.net/). A CC was defined as a group of isolates that shared at least six of seven alleles with another ST in the group [29]. Pairs of isolates that differ at only one of the seven MLST loci were called single-locus variants (SLVs) [29].

### Phylogenetic analysis

A maximum likelihood (ML) phylogenetic tree was constructed from the nucleotide sequence of concatenated MLST genes, after alignment using ClustalW using MEGA 6 [30]. Default parameters were used for analysis and phylogenetic tree construction.

### Genetic diversity

The genetic diversity, as revealed by PFGE, was calculated using the Simpson’s Index of Diversity (SID) as previously described [31] with the freely available online tool Comparing Partitions (http://darwin.phyloviz.net/Comparing Partitions/index.php?link = Home). The 95% confidence intervals (95% CI) were also calculated.

### Estimates of recombination rates

The per-allele and per-site recombination to mutation (r/m) rates were empirically estimated through the calculation of the number of polymorphisms arisen by recombination or mutation as previously described [32]. Briefly, the sequences of the alleles that differ between each ancestral ST and its associated SLVs are compared and are assigned as resulting from either a recombination or a point mutation. The emergence of the variant allele was considered as having simply arisen by point mutation, if the variant allele in one isolate differed at a single nucleotide site from the corresponding allele in the descendant SLV. However, the emergence of the variant allele was considered to have likely resulted from a recombination event, if the difference involved multiple nucleotide changes or single-nucleotide change previously observed within the collection analyzed.

### Stability assays *in vitro*

#### Genotypic assays

To evaluate the stability of IS*1272* and PFGE profiles, two strains (HSM742 and ITL578) belonging to PFGE types with the highest number of subtypes (PFGE C and D) were used. The two strains selected harbored SCC*mec* V and belonged to different STs (ST1 and ST3, respectively). Isolates were subjected to serial passages on tryptic soy broth (TSB). A single colony was transferred to 5 mL of TSB and grown 24 hours at 37°C. Cultures were daily transferred to fresh liquid medium (1:100 dilution) followed by growth during 24 h at 37°C with aeration for a total period of 34 days. Cultures were frozen every day during the 34 days. Agarose disks containing chromosomal DNA were prepared from a 500 μl aliquot of the liquid cultures corresponding to days 0, 4, 7, 10, 13, 16, 19, 22, 25, 28, 31 and 34 and stored at 4°C. PFGE was performed for the descendants and their parental strains and the resultant SmaI profiles were hybridized with a probe specific for IS*1*272. An unrelated MRSA isolate *S*. *aureus* WIS was used as an internal control for the stability assay reproducibility. The study was carried out following the consensus guidelines for appropriate use and evaluation of microbial epidemiological typing systems [33].

The stability of SCC*mec* during prolonged serial subculture was investigated by direct PCR amplification of the *mec* complex C2 and *ccrC* [24,34] from different colonies (http://2013.igem.org/Team:Penn). Cultures corresponding to day 0, day 25 and day 28 were plated (1:50 dilution) and a total of 10 colonies for each day were picked up from agar plates by using a sterile pipette tip and resuspended into 20 μl of sterile MilliQ water. 1 μl of this suspension was used as a template in a 50 μl PCR reaction mix. The 30 PCR cycles (94°C for 30 s, 56°C for 1 min, and 72°C for 2 min) were preceded by heating to 95°C for 6 min for initial cell breakage and DNA denaturation and followed by a 10-min incubation at 72°C. *S*. *aureus* WIS was used as a positive control for the PCR.

#### Phenotypic assays

The in vitro stability of antimicrobial resistance and virulence phenotypes was assessed by comparing the oxacillin and cefoxitin minimum inhibitory concentrations, hemolysis, mannitol and biofilm production of the parental strains and their descendants after serial passage *in vitro*. The oxacillin and cefoxitin MICs were determined by E-test (AB BioMérieux, Solna, Sweden). Biofilm production was performed by the microtiter plate assay method in TSB and in TSB supplemented with 1% glucose (TSBglu) [3,35]. Hemolysis was tested by spotting 5 μl drops of an overnight culture suspension, adjusted to 0.5 McFarland standard, onto filter paper discs (Becton, Dickinson and Company, Le Pont de Claix, France) properly deposited over the surface of blood agar plates [36] for 24h and 48h incubation at 37°C. For mannitol fermentation ability testing, isolates were cultured on mannitol salt agar plates (Becton, Dickinson and Company, Le Pont de Claix, France) for 24 and 48h incubation at 37°C.

## Results

### Antibiotic susceptibility patterns

The results of susceptibility to a panel of 14 antibiotics are shown in S1 Table. A total of 130 out of 133 isolates (98%) expressed phenotypic methicillin resistance, with 71% showing a high-level resistance to oxacillin (MIC>256 μg/ml). However, three isolates lacked *mecA*, in spite of the fact they were resistant to oxacillin and cefoxitin in disc diffusion and to oxacillin in E-test (MICs 12–64 μg/ml); this discrepancy was not investigated further.

All MRSHae isolates were resistant to at least one of the non-β-lactams antibiotics tested and showed high rates of resistance to gentamicin (106 isolates; 83.5%), erythromycin (103; 81%), trimethoprim-sulfamethoxazole and ciprofloxacin (96; 75.6% each), tetracycline (56; 44%), chloramphenicol and clindamycin (37; 29% each). The resistance rate to rifampin was low (12; 9.5%). Although there were no isolates resistant to quinupristin-dalfopristin and vancomycin, 19% were resistant to teicoplanin according to CLSI criteria [18]. Interestingly, 88% of MRSHae isolates showed multiresistance which was not exclusively associated with methicillin-resistant isolates: the few MSSHae (n = 6) were found to be also multidrug-resistant (S1 Table).

### Genotypic analysis

The molecular characterization of isolates by SCC*mec* typing showed that the majority of the MRSHae population (65.4%) carried new or non-typeable SCC*mec* types. Only a small proportion of the population (44 isolates, 34.6%) could be assigned to known SCC*mec* types, namely types I, III, IV, and V. SCC*mec* type V (5C) was the most frequent, being identified in 28% of the collection analyzed (36 isolates). The remaining SCC*mec* types were present at very low frequency, including types I and III (1 isolate, 0.8% each) and IV (6, 4.7%).

PFGE analysis classified the 127 out of 133 MRSHae isolates into 26 different PFGE types (A-Z) and 123 subtypes (S1 Table) revealing an extremely high genetic diversity in the genetic background [SID = 0.933 (95 CI, 0.912–0.953) for the PFGE type and SID = 0.996 (95 CI, 0.994–0.999) for the PFGE subtype]. The majority of isolates (57/127, 45%) were clustered in four major PFGE types (A, B, C, and D). PFGE type A was found to be the predominant, containing 23 isolates (18%), followed by type B (13 isolates, 10%), C (11 isolates, 8.7%), and D (10 isolates, 7.8%). The remaining isolates belonged to 22 scarcely represented PFGE types (S1 Table).

The 6/133 MSSHae isolates were classified into three PFGE types and six subtypes (S1 Table). Three out of the six MSShae strains had PFGE macrorestriction profiles that were related (similarity> 78%) with those found in MRSHae.

**Fig 1:**
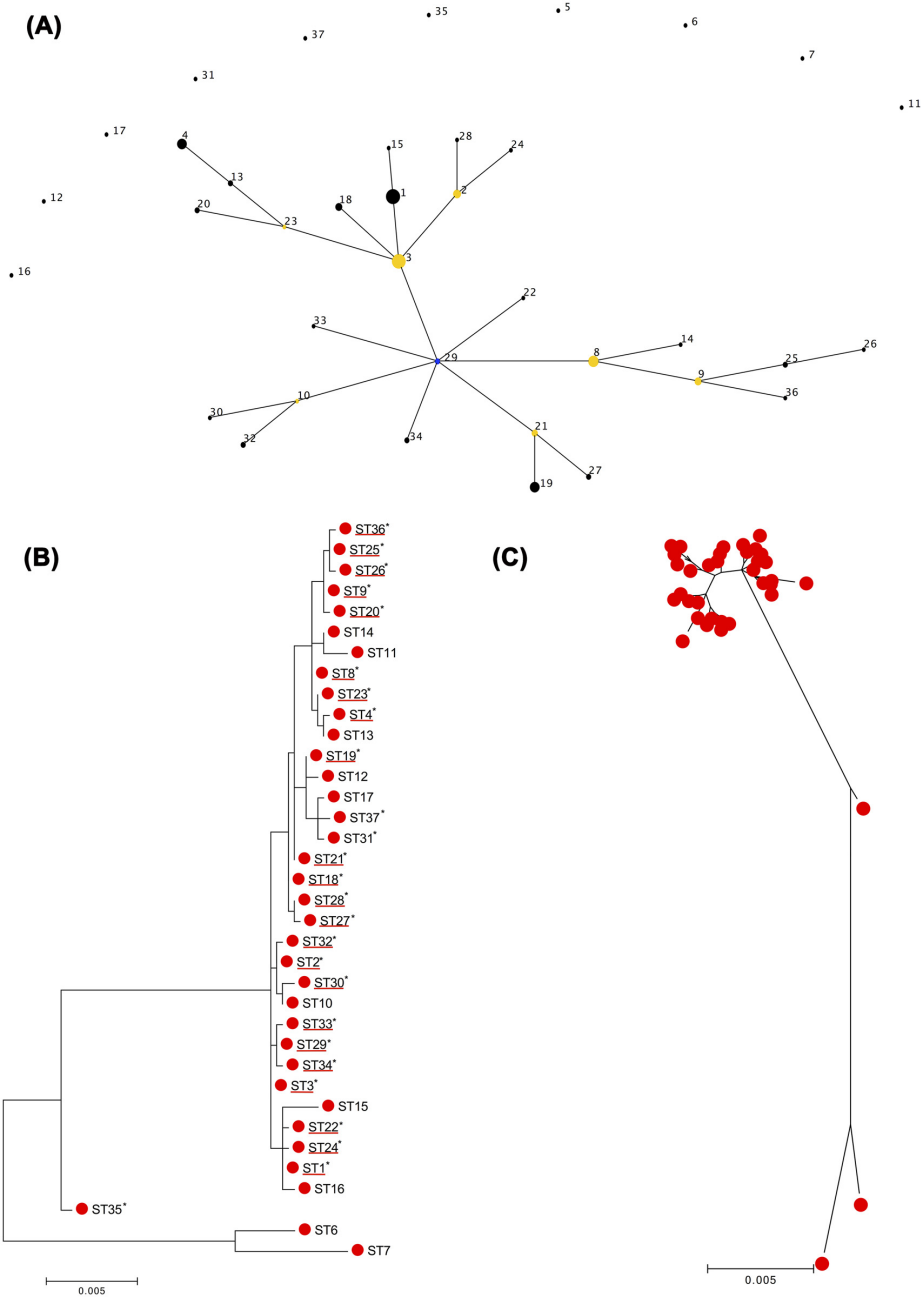
Phylogenetic analysis. (A) Identification and characterization of a major genetic lineage. Application of eBURST algorithm to MLST data for the collection of 109 *S*. *haemolyticus* isolates obtained in this study and that of Cavanagh [11]. The relatedness between STs and clonal complexes was displayed as an eBURST diagram. Each ST is represented by a number and a node, and each line links STs that are single locus variants (SLVs). Bleu node corresponds to the founder (ST29). The size of the node corresponds to the frequency of the isolates. Clusters of linked STs correspond to clonal complexes. (B-C) Phylogenetic trees inferred from the concatenated sequences of the seven MLST loci. Maximum likelihood (ML) Phylogenetic tree was constructed based on concatenated sequences of 7 housekeeping loci for 26 STs obtained in the present study and 17 STs identified in Cavanagh study [11]. The trees were drawn to scale using MEGA 6. The ML analysis identified two groups one containing the great majority of STs that corresponded to CC29 identified by eBURST analysis, and the other containing only four STs. Asterisks indicate the STs identified in this study. STs of CC29 are underlined with red. Each red circle on phylogenetic tree (C) corresponds to an ST [The same ST in ML tree (B)].

The molecular characterization of 65 *S*. *haemolyticus* isolates, representative of all PFGE subtypes-SCC*mec* combinations, by MLST identified 26 different sequence types (STs) (ST1-ST4, ST8-ST9, ST18-ST37), indicating a high level of genetic diversity (Table 1, Fig 1). A total of 20 STs and seven new gene alleles were identified in this study. Sequences of the new alleles have been submitted to GenBank with the following accession numbers: KX232467-KX232473. The most prevalent type was ST3 (9 isolates, 14%), followed by ST19 (8 isolates, 12%), ST4 (5 isolates, 7.7%), ST1, ST8 and ST18 (4 isolates each, 6%), ST2 and ST21 (3 isolates, 4.6%) and ST9, ST20, ST25, ST27, ST29, ST32, ST34 (2 isolates each, 3%).

**Table 1:** Molecular characterization of 65 MRSHae and MSSHae isolates.

Clonal complex (CC) or Singleton (S)	Country (No)	ST (No)	PFGE type (No)	SCC*mec* (No)	Number of IS copies (No)
S31	CHI (1)	31 (1)	P1 (1)	V (1)	5 (1)
S35	ITL (1)	36 (1)	F2 (1)	NT (1)	8 (1)
S37	ITL (1)	38 (1)	B1 (1)	NT (1)	6 (1)
CC29	MCO (1), PTL (3)	1 (4)	M1 (1), O1 (1), O2 (1), C7 (1)	IV (1), V (2), NT (1)	7 (3), 9 (1)
	HUR (1), ITL (1), PLN (1)	2 (3)	I3 (1), Q2 (1), 03 (1)	V (1), NT (2)	7 (1), 8 (1), 10 (1)
	BUG (1), CHI (2), CHL (1), ITL (2), PTL (3)	3 (9)	B1 (1), D6 (1), E3 (1), I4 (1), J1 (1), M3 (1), Q1 (1), S1 (1), Y (1)	V (5), NT (4)	3 (1), 6 (1), 7 (3), 8 (2), 10 (2)
	CHI (1), DEN (2), ICE (1), TAW (1)	4 (5)	A1 (1), A5 (1), A23 (1), E1 (1), L1 (1)	NT (5)	7 (1), 9 (1), 11 (3)
	DEN (1), MCO (1), URU (1), ITL (1)	8 (4)	N1 (1), N2 (1), N3 (1), C (1)	I (1), IV (1), V (1), MSSHae (1)	7 (3), 8 (1)
	CHI (2)	9 (2)	G1 (2)	V (1), NT (1)	8 (2)
	AGT (1), CHI (1), DEN (1), PTL (1)	18 (4)	A20 (1), D8 (1), S2 (1), V1 (1)	NT (4)	7 (3), 11 (1)
	AGT (1), ITL (2), JAP (1), PLN (1), CHI (3)	19 (8)	A1 (1), A2 (1), A3 (1), B2 (1), F6 (1), H1 (1), R2 (1), Z (1)	NT (4), MSSHae (4)	1 (1), 4 (1), 5 (1), 6 (2), 7 (1), 9 (1), 10 (1)
	BUG (1), ICE (1)	20 (2)	C1 (1), K4 (1)	NT (2)	6 (1), 8 (1)
	CHI (3)	21 (3)	R1 (1), U1 (1), U2 (1)	NT (3)	8 (3)
	CHL (1)	22 (1)	J1 (1)	V (1)	8 (1)
	HUR (1)	23 (1)	I1 (1)	NT (1)	9 (1)
	ICE (1)	24 (1)	A9 (1)	NT (1)	8 (1)
	ICE (1), ITL (1)	25 (2)	F5 (1), T2 (1)	IV (1), NT (1)	7 (1), 9 (1)
	ITL (1)	26 (1)	T1 (1)	V (1)	8 (1)
	ITL (2)	27 (2)	D1 (1), E3 (1)	IV (1), NT (1)	9 (2)
	ITL (1)	28 (1)	J5 (1)	NT (1)	8 (1)
	MCO (1), PTL (1)	29 (2)	A12 (1), P2 (1)	V (1), NT (1)	9 (2)
	URU (1)	30 (1)	H4 (1)	V (1)	5 (1)
	BUG (1), CHI (1)	32 (2)	K1 (1), X (1)	V (1), NT (1)	7 (1), 8 (1)
	CHI (1)	33 (1)	K2 (1)	V (1)	10 (1)
	ICE (1)	34 (2)	C5 (1), C6 (1)	NT (2)	9 (1), 10 (1)
	PLN (1)	36 (1)	G3 (1)	MSSHae (1)	7 (1)

No, Number of isolates; CHI, China; ITL, Italy; MCO, Mexico; PTL, Portugal; HUR, Hungary; PLN, Poland; BUG, Bulgaria; CHL, Chile; DEN, Denmark; ICE, Iceland; TAW, Taiwan; URU, Uruguay; AGT, Argentina; JAP, Japan; ND, not determined; ST, sequence type; NT, non-typeable; IS, insertion sequence.

The remaining 12 STs were detected in single isolates only. Of the 65 *S*. *haemolyticus* tested, we could not assigne an ST to three isolates due to failure of the sequencing reaction of the *SH1431* and *hemH* genes.

As previously observed [11], no good correlation was found between ST and PFGE types (Table 1, S1 Table). Several isolates with identical STs belonged to different pulsotypes and conversely isolates with the same PFGE pulsotype belonged to different STs. In particular, in seventeen situations, isolates assigned to the same PFGE type belonged to different STs. However, in fourteen of these situations, the STs belonged to the same clonal complex (CC29). On the other hand, the PFGE types N and U included isolates that were assigned to the same ST8 and ST21, respectively. One interesting observation was the fact that the most common and disseminated STs (ST3 and ST19) were the ones containing the highest number of PFGE types and subtypes.

### Phylogenetic analysis

The analysis of MLST data by eBURST algorithm, including the STs identified in this study and STs previously identified [11], showed that *S*. *haemolyticus* population was composed of a major clonal complex comprising as many as 27 different STs, in which ST29 was identified as the founder, and 10 singletons (ST5, -6, -7, -11, -12, -16, -17, -31, -35, -37).

In order to validate the clustering and evolutionary model generated by eBURST, an ML tree was reconstructed from the concatenated nucleotide sequences of seven MLST loci for the 26 STs obtained in this study and the 17 STs identified in Cavanagh study [11]. The ML resulting tree separated the isolates in only two groups one containing the great majority of isolates and STs and the other containing only four sequence types (ST6, ST7 and ST35).

Globally, eBURST and ML tree analysis were consistent with one another. The different groups found by eBURST also grouped together in the ML tree. However, the ML tree gave better resolution by revealing some phylogenetic relationships among singletons not observed by eBURST (Fig 1A and 1B). For example ST11, ST12, ST16, ST17, ST31 and ST37 that according to eBURST analysis were separated from the major group, were found to be closely related to the major cluster in the ML tree. Additionally, the ML tree showed that ST12, ST17, ST31 and ST37 (ST17 and ST31 or ST37 were DLVs) were closely related, a relatedness also not detected by eBURST. A similar case was observed for ST11 and ST14 (TLVs) and for ST1 and ST16 (SLVs) that were closely related in the ML tree.

### Distribution of SCC*mec* among *S*. *haemolyticus* population

The combination of MLST and SCC*mec* typing data showed that SCC*mec* type V was present in several distinct clonal types, including twelve STs (ST1, ST2, ST3, ST8, ST9, ST22, ST26, ST29, ST30, ST31, ST32 and ST33), most of them belonging to CC29 (Table 1, Fig 1). Moreover, isolates with identical STs carried more than one SCC*mec* type (ST1 with SCC*mec* types V and IV and ST8 with SCC*mec* types I, V, and VI), suggesting that the SCC*mec* acquisition occurred in more than one occasion. Considering the relatedness of STs as revealed by eBURST and SCC*mec* typing data and using the method previously described [27,28], we estimated that overall, SCC*mec* was acquired at least 14 times by *S*. *haemolyticus*. However, given the high number of non-typeable SCC*mec*, which were not taken into consideration in this estimation, it is probable that this number is larger.

### Contribution of mutation and recombination to clonal diversification

To understand the mechanisms contributing to genetic diversity in *S*. *haemolyticus* we estimated the rates of recombination and mutation through the analysis of MLST data. The comparison of the nucleotide sequence of each variant allele with the corresponding allele in the descendant SLV showed that 14 out of the 26 single-locus variations occurring in our collection originated from a recombination event and 13 arose by mutation. The number of variant alleles that have arisen by recombination compared to point mutation resulted in a per-allele r/m parameter of approximately 1:1 and a per site r/m rate of 2.9:1. This implies that for each allele changed by mutation; approximately one is changed by recombination and that the probability of each site of evolving by recombination is three times greater than that of evolving by mutation. Thus, both recombination and mutation appear to be significant factors in structuring the *S*. *haemolyticus* population.

### Frequency and distribution of IS*1272* insertion sequence

Although *S*. *haemolyticus* carries different IS elements, in this study, we chose to investigate the distribution of IS*1272*, which has been described previously to be the most prevalent [4]. As a screening method for the presence of IS*1272* and estimation of copy number, we chose to hybridize SmaI DNA patterns with IS1272 probe. Actually, virtually all isolates (132 out of 133 isolates) carried the IS*1272* insertion sequence as determined by Southern hybridization, although they varied in the number of IS*1272*-hybridizing bands. Because IS*1272* does not contain a SmaI site, the number of IS was roughly estimated for each isolate by counting the number of IS*1272*-hybridizing bands. However, we cannot exclude the hypothesis that high-molecular-weight bands may contain more than one copy of the IS and consequently this counting may represent a low estimate of the number of IS*1272* copies.

Hybridization with IS*1272* produced multiple bands in all isolates except one that showed a single IS*1272* hybridization band. Each of the isolates examined that showed multiple bands had between 3 and 13 IS*1272*-hybridizing fragments, of sizes varying from 48.5 and 485 Kb, indicating that the element is inserted throughout the genome. The number of IS copies showed a Gaussian distribution, the great majority of strains (59%) carried between 7–8 copies. Only 3% of the strains carried a number of copies that was equal or lower than 4 and less than 1% had more than 11 IS*1272* copies in the chromosome. Both MSSHae isolates and MRSHae had multiple IS*1272*-hybrization bands but MRSHae had a wider range of IS*1272* copy number (1–13 copies) than MSSHae (4–9 copies). In particular, the most prevalent STs identified among MRSHae (ST3 and ST19), had the widest range of IS copies, suggesting the existence of a high rate of transposition, and coincidently had also the highest number of PFGE subtypes. However no correlation was found between the number of IS*1272* hybridization bands and the existence of a multidrug resistant profile (Table 1, S1 Table).

### Stability of IS*1272* and PFGE profiles *in vitro*

**Fig 2:**
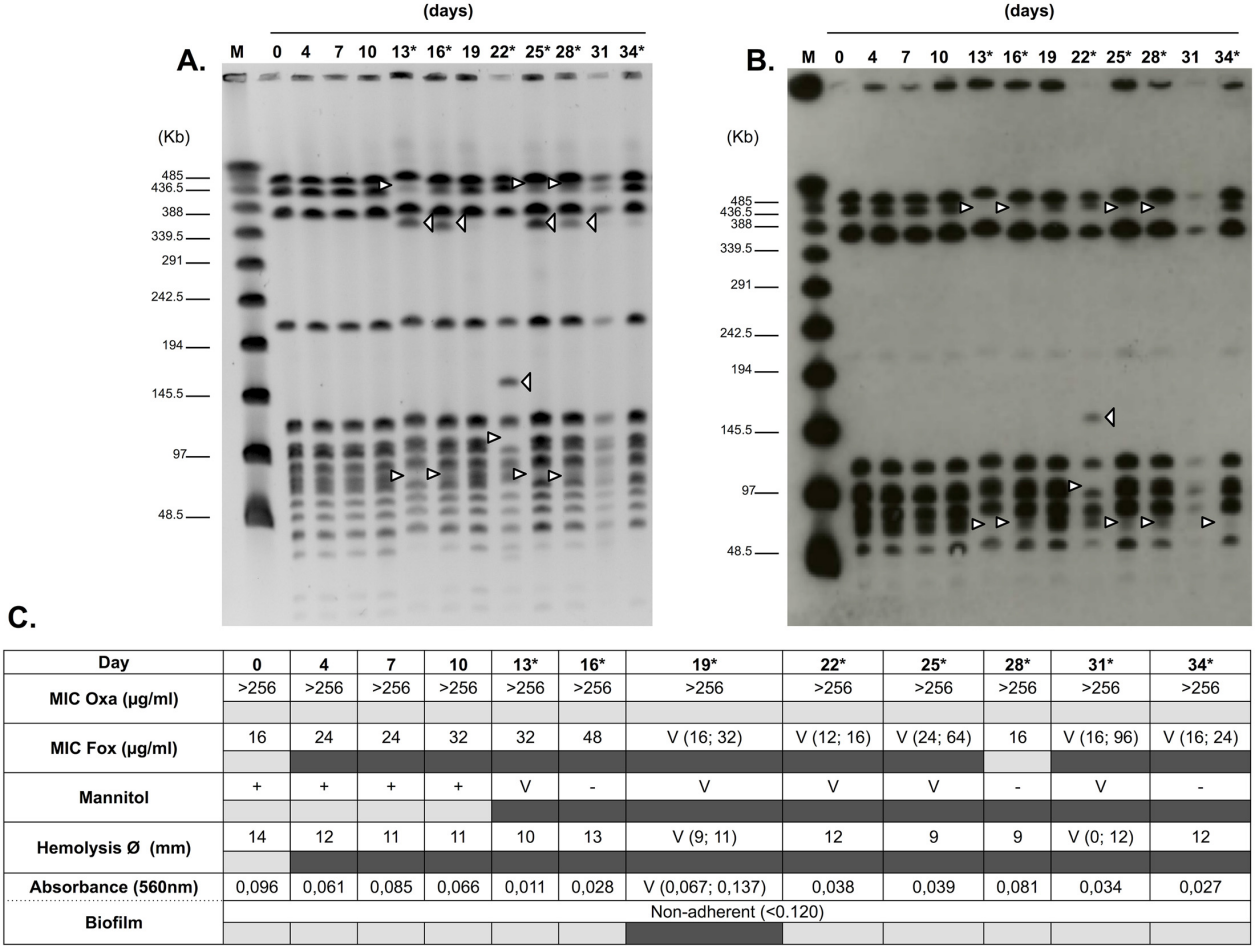
Phenotypic and genotypic *In vitro* stability. (A) *In vitro* stability over time of SmaI PFGE profiles. (B) *In vitro* stability over time of IS*1272*-hybridization patterns. Lane M: λ ladder used as a size marker. The positions of molecular markers (in kilobase pairs) are indicated by black lines. The white arrows indicates band loss/gain in PFGE and SmaI-IS*1272* profiles. The arrowheads on the right of each lane correspond to the appearance of a band. The arrowheads on the left of each lane correspond to the disappearance of a band. At the top of each lane the day that the MRSHae HSM742 isolate was collected is indicated in relation to the first isolate (day 0). (C) Stability over time of phenotypic traits. Oxa, oxacillin; Fox, cefoxitin; empty set (Ø), hemolysis zone diameter; a plus sign indicates fermentation of mannitol; a minus sign indicates no fermentation of mannitol; V, variable. The asterisks indicate days that showed alteration. The presence (dark gray) and absence (light gray) of alteration are indicated by filled rectangles.

The stability of IS*1272* copy number and chromosomal position as well as the stability of PFGE-SmaI patterns was determined over time by following serial growth *in vitro* of two strains belonging to PFGE types with the highest number of subtypes (PFGE C and D). A high instability of both SmaI PFGE patterns and IS*1272* hybridization patterns was observed in the two strains tested. The SmaI patterns and IS*1272* hybridization patterns of the descendant strains were checked every 3 days of growth and along the 34 days of passage the patterns changed several times in two or more bands and in some occasions reverted to the original genotype. The PFGE patterns of *S*. *haemolyticus* strain HSM742 from day 0 was indistinguishable from those obtained on days 4, 7, 10, 19 and 31; however, the PFGE of strains isolated in days 13, 25 and 28 gained, each, one band with identical position (approximate molecular weight 358 Kb) and lost two bands (approximate molecular weights 432 and 74 Kb) relatively to the ancestral strain; the PFGE pattern of strain isolated in day 16 lost a 74-Kb band and gained a 432 band; the PFGE profile of strain isolated on day 22 gained an additional band of 159 Kb and lost one band of 108 Kb (Fig 2A). In some PFGE macrorestriction patterns faint bands were observed that suggest the simultaneous existence of different subpopulations, indicating that band loss and gain probably occurred only in part of the population.

Most of the changes in the SmaI-IS*1272* patterns paralleled those observed in SmaI-PFGE profiles (Fig 2B)—like in SmaI patterns, in IS*1272* SmaI hybridization pattern we observed the loss of 432-Kb and 74-Kb fragments in isolates from days 13, 25 and 28 and the loss of 74-Kb fragment and gain of 432 Kb band on day 16. Likewise we observed the gain of a159Kb band and the loss of 108 Kb band in strain isolated in day 22. The only discrepancies observed between the PFGE and SmaI-IS*1272* patterns regard to the acquisition of the 358 kb SmaI band in the PFGE patterns of strains isolated in days 13, 16, 25 and 28 and the acquisition of the 74 Kb SmaI-IS*1272* hybridization band of strain isolated in day 34. Also, we observed that the banding patterns of isolates collected in days 13, 16, 25 and 28 showed differences in band intensity, which might correspond to the existence of subpopulations. The most straightforward explanation for the discrepancies observed between the PFGE and SmaI-IS1272 hybridization patterns is the involvement of IS elements other than IS*1272*, which were not tested here. Also, subpopulations with low representativeness in the overall population might be missed using this method.

In spite of the discrepancies observed, overall, the results suggest that instability of chromosome was highly associated with acquisition and loss IS*1272* elements.

### Stability of phenotypic traits *in vitro*

In order to understand if genotypic changes observed in SmaI PFGE macrorestriction patterns and SmaI-IS*1272* hybridization patterns had impact on clinically important phenotypic traits we additionally characterized the cultures after serial growth *in vitro* for susceptibility to beta-lactams, mannitol fermentation, hemolysis, biofilm formation and morphology.

At the phenotypic level, the HSM742 strain showed maintenance of high-level oxacillin-resistance (MIC>256 μg/ml) despite serial passage on the TSB medium. However, the MICs for cefoxitin suffered several changes over time. The MIC for cefoxitin has triplicated in the first 16 days of serial passage *in vitro* and after that, until day 34, showed a variable phenotype, wherein some colonies within the same culture had different levels of resistance (Fig 2C).

Phenotypic changes were also observed along time for mannitol fermentation, hemolysis and biofilm formation. After day 10, mannitol changed from positive to variable or negative in the following days. Also after day 0, the level of hemolysis, as determined by the halo diameter formed around drop of culture suspension, decreased or became variable. The absence of hemolysis was even detected in day 31 (diameter = 0 mm). Regarding Biofilm production, the strain changed from being a biofilm non-producer to a biofilm producer on day 19 (A560>0.120). No significant differences in colonial morphology were noted when the MRSHae isolate was serially passaged and compared to the original isolate.

Although it was difficult to make a direct association between the changes observed in the PFGE and SmaI-IS*1272* patterns with the phenotypic changes observed, serial passage created instability both in genotypic and phenotypic features in the *S*. *haemolyticus* strains tested. Moreover, phenotypic variability was more frequent after day 10, wherein alterations in genotypic traits were also observed.

### Stability of SCC*mec in vitro*

We noticed that a high number of non-typeable and new SCC*mec* types were identified in the *S*. *haemolyticus* collection analyzed. To understand if SCC*mec* structure was also unstable during growth *in vitro* the parental strains and the variants obtained after serial growth *in vitro* (10 colonies each) were tested for the presence of *mecA* by Southern hybridization and screened for changes in SCC*mec* structure by PCR. No loss of SCC*mec* during growth neither SCC*mec* structural changes were observed in any of the isolates examined, suggesting the stability of SCC*mec* in the conditions tested.

## Discussion

In the present study we gained a better understanding of the molecular epidemiology and mechanisms of evolution of *S*. *haemolyticus* in the hospital environment by analysis of a geographically diverse collection of nosocomial *S*. *haemolyticus* by a combination of genotypic and phenotypic methods.

The analysis of *S*. *haemolyticus* collection by MLST has identified a single worldwide disseminated prevalent genetic lineage of nosocomial *S*. *haemolyticus*, designated CC29, which accounted for 91% of the nosocomial *S*. *haemolyticus* analyzed. This genetic lineage was highly diverse comprising as many as 27 different STs. A previous study by Cavanagh *et al*. [11] where nosocomial isolates were also characterized by MLST, have revealed the existence of three different CCs in the nosocomial population. However, the addition of MLST data from our study to the previously provided MLST data led to the merging of those three groups into a single major clonal complex. This major CC29 probably corresponds to the major group previously identified by whole genome sequencing analysis of nosocomial *S*. *haemolyticus* [12] that included only isolates from Europe. Our study revealed, however, that this major clonal complex was not exclusively found in Europe, but was also disseminated in Asia and South America. This type of population structure where a single major CC complex includes the great majority of the nososcomial isolates independently of geographic origin is very similar to that described for *S*. *epidermidis*, another coagulase-negative staphylococcal species that shares the same ecological niche [28], suggesting that a similar strategy may be used by both species to adapt to host during hospitalization.

Like it was found before for *S*. *epidermidis* [28], we discovered that also in *S*. *haemolyticus*, homologous recombination contributes significantly for evolution. The *S*. *haemolyticus* per allele r/m rates estimated in this study (1:1) showed that recombination had a higher impact in this species than in *S*. *aureus* (1:2) [37], however, does not reach the levels of recombination observed in *S*. *epidermidis* (2.5:1) [28]. Although the occurrence of homologous recombination was previously observed to occur in *S*. *haemolyticus* [12], this is the first study wherein the extension of such phenomena in *S*. *haemolyticus* population was addressed. The existence of such a big and diverse clonal complex in *S*. *haemolyticus* may in fact derive from the frequent occurrence of homologous recombination in housekeeping genes that are distributed along the chromosome, which is accordance with the fact that recombination was found to arise in diverse genetic regions, which span around 78% of the chromosome [12]. However, recombination events based on the seven MLST alleles may not always represent what happens throughout the entire genome.

Another mechanism identified in this study contributing to the genetic diversity and to the shaping of *S*. *haemolyticus* population structure is the IS-mediated chromosomal rearrangements. We found that the great majority of nosocomial *S*. *haemolyticus* carried a high number of IS*1272* copies (3–13 copies), as determined by IS*1272*-SmaI fragments hybridization. However, this number is lower than that estimated by Archer *et al*. [38], who identified 20 or more copies of IS*1272* in *S*. *haemolyticus* isolates, however the method used was different. This difference might be related with the methodology used or the type of sample analyzed. Moreover, we found that the *S*. *haemolyticus* chromosome of one of the most frequent clonal types in our collection (ST1) was highly unstable during serial growth *in vitro*, which paralleled with the instability of the IS-SmaI patterns. These results suggest that transposition or recombination between IS copies might be promoting frequent chromosome rearrangements, as previously observed [4]. The occurrence of frequent IS-mediated chromosome rearrangements within isolates of a single ST might give rise to diverse SmaI macrorestriction patterns, like we observed in this study to occur in ST19 and ST3. This phenomenon might lead to the loss of phylogenetic signal in PFGE profiles and may be an explanation for the lack of correlation between MLST and PFGE in the *S*. *haemolyticus*.

Genetic diversity in SCC*mec* carried by *S*. *haemolyticus* was also apparent in our collection and was repeatedly described in the literature [22,39–41]. Particularly impressing is the high number of non-typeable SCC*mec* types described in this species, which in our collection accounted for 65.4% of the population. Recombination was previously reported to be very frequent within the genomic region around the origin of replication, the so-called *oriC* environ [4,12], wherein SCC*mec* is inserted, and IS were also found to be frequent. It is tempting to speculate that the high number of non-typeable SCC*mec* found in *S*. *haemolyticus* results from SCC*mec* rearrangements promoted by recombination and IS-induced genetic rearrangements. However, during the stability assays performed no SCC*mec* structural variations were observed by the methods used, suggesting other mechanism may be involved or that particular environmental signals, not tested in this study, may trigger SCC*mec* variation. Actually, the existence in our collection of six methicillin-susceptible *S*. *haemolyticus* strains, all with a multidrug resistant profile, suggest that recombination involving cassette chromosome recombinases and *mecA* deletion, as previously documented [42], may occur relatively frequently in *S*. *haemolyticus* and may be the main mechanism of variation within SCC*mec*.

Besides promoting genetic variation we observed that IS movement and/or chromosomal alterations during stability assays also paralleled alterations in phenotypic features which can be clinically significant, namely in mannitol fermentation, hemolysis and biofilm formation, most of which were reversible. The reversible nature of this phenomena, suggest that phenotypic changes observed might be generated by transposition of IS*1272* that can alter gene expression either by disruption of genes or by insertion in gene promoters [4,13], but that can similarly be excised leaving genes and promoters intact and reverting the phenotype. The tuning of these functions might be important in the change from commensal to a pathogen lifestyle. Further study based on whole genome sequencing would be required to clarify this issue and to define the causal mechanism.

Our findings suggest that IS-mediated chromosomal rearrangements and recombination had a crucial role in shaping the population structure and evolution of *S*. *haemolyicus* and play an important role in genome flexibility and adaption to host and hospital environment.

## Supporting Information

S1 Table Epidemiological data and molecular characterization of all 133 tested *S*. *haemolyticus* isolates. (DOC)
