# Supplementary material for: Impact of Insertion Sequences and Recombination on the Population Structure of Staphylococcus haemolyticus
Source: PLoS One. 2016 Jun 1;11(6):e0156653. doi: 10.1371/journal.pone.0156653 (PMC4889114; doi:10.1371/journal.pone.0156653)
Supplement: S1 Table — (DOC) [file pone.0156653.s001.doc]

**Table S1. Epidemiological data and molecular characterization of all 133 tested *S. haemolyticus* isolates.**

| **Strain** | **Geographic location** | **Year of isolation** | **Source** | **Origin** | **MIC OXA (µg/ml)** | ***mecA*** | **SCC*mec* type** | **PFGE type** | **ST** | **Number of *IS*1272 copies** | **Resistance profile** |
| --- | --- | --- | --- | --- | --- | --- | --- | --- | --- | --- | --- |
| AGT145 | Argentina | 1998 | Wound | Infection | >256 | Positive | NT | D8 | 18 | 7 | PEN, OXA, CFX, SXT, CIP, CHL, CLN, ERY, GEN, RIF, TET, TE |
| AGT149 | Argentina | 1998 | Wound | Infection | >256 | Positive | NT | F6 | 19 | 7 | PEN, OXA, CFX, SXT, CIP, CLN, ERY, GEN |
| BRA181 | Brazil | 1998 | Blood | Infection | 32 | Positive | V | I5 | ND | 7 | PEN, OXA, CFX, SXT, CIP, CHL, CLN, GEN, TET |
| BUG70 | Bulgaria | 1998 | NP | Infection | 1 | Positive | V | G3 | ND | 8 | PEN, OXA, CFX, TET |
| BUG72 | Bulgaria | 1998 | NP | Colonisation | 1 | Positive | NT | K1 | 32 | 7 | PEN, OXA, CFX, SXT, CHL, ERY |
| BUG78 | Bulgaria | 1998 | NP | Infection | >256 | Positive | NT | K4 | 20 | 8 | PEN, OXA, CFX, SXT, CIP, CHL, CLN, ERY, GEN, TET |
| BUG98 | Bulgaria | 1998 | NP | Colonisation | 32 | Positive | V | G5 | ND | 7 | PEN, OXA, CFX, SXT, GEN, TET, TE |
| BUG106 | Bulgaria | 1998 | Wound | Colonisation | >256 | Positive | V | F1 | NT | 6 | PEN, OXA, CFX, SXT, CHL, ERY, GEN, TET |
| BUG119 | Bulgaria | 1998 | NP | Colonisation | >256 | Positive | V | S1 | 3 | 10 | PEN, OXA, CFX, SXT, CIP, ERY, GEN, RIF, TET |
| CHI49 | China | 1998 | Respiratory | NP | >256 | Positive | NT | R1 | 21 | 8 | PEN, OXA, CFX, SXT, CIP, CHL, ERY, GEN, TET |
| CHI51 | China | 1998 | Blood | Infection | >256 | Positive | NT | F3 | ND | 8 | PEN, OXA, CFX, SXT, ERY, GEN, RIF |
| CHI52 | China | 1998 | Urine | Infection | 0.75 | Positive | V | P1 | 31 | 5 | PEN, OXA, CFX, TET |
| CHI54 | China | 1998 | Wound | Infection | 48 | Positive | V | P3 | ND | 8 | PEN, OXA, CFX, SXT, CIP, ERY, GEN, TET |
| CHI57 | China | 1998 | Respiratory | NP | >256 | Positive | NT | L1 | 4 | 11 | PEN, OXA, CFX, SXT, CLN, ERY, TET |
| CHI60 | China | 1998 | Abces | NP | 48 | Positive | V | Q1 | 3 | 6 | PEN, OXA, CFX, SXT, CIP, CHL, CLN, ERY, GEN, TET, TE |
| CHI69 | China | 1998 | Respiratory | NP | 0.38 | Positive | V | G1 | 9 | 8 | PEN, OXA, CFX, CIP, GEN, TET |
| CHI72 | China | 1998 | Abces | Infection | >256 | Positive | V | H5 | ND | 9 | PEN, OXA, CFX, SXT, CIP, ERY, GEN |
| CHI74 | China | 1998 | Blood | Infection | 2 | Positive | V | K2 | 33 | 10 | PEN, OXA, CFX, CLN, ERY |
| CHI76 | China | 1998 | Respiratory | Infection | 0.5 | Positive | V | K3 | ND | 7 | PEN, OXA, CFX, SXT, CLN, ERY, GEN, TET |
| CHI80 | China | 1998 | Urine | Infection | 0.38 | Positive | NT | S2 | 18 | 7 | PEN, OXA, CFX, SXT, CIP, ERY, TET |
| CHI81 | China | 1998 | Urine | Infection | 32 | Positive | V | M3 | 3 | 7 | PEN, OXA, CFX, CIP, ERY, GEN, TET |
| CHI82 | China | 1998 | Respiratory | Infection | 1.5 | Positive | NT | U1 | 21 | 8 | PEN, OXA, CFX, CIP, GEN, TET |
| CHI83 | China | 1998 | Abces | Infection | 12 | Positive | NT | G1 | 9 | 8 | PEN, OXA, CFX, CIP, GEN, TET |
| CHI88 | China | 1998 | Respiratory | Infection | >256 | Positive | V | X | 32 | 8 | PEN, OXA, CFX, SXT, CIP, CHL, ERY, GEN, TET |
| CHI89 | China | 1998 | Respiratory | Infection | 1 | Positive | NT | U2 | 21 | 8 | PEN, OXA, CFX, CIP, CHL, ERY, GEN, TET |
| CHL83 | Chile | 1998 | NP | NP | 12 | Positive | V | J1 | 22 | 8 | PEN, OXA, CFX, SXT, CHL, ERY, GEN, TET |
| CHL157 | Chile | 1998 | NP | NP | 192 | Positive | V | J1 | ND | 8 | PEN, OXA, CFX, SXT, CHL, ERY, GEN, TET |
| CHL160 | Chile | 1998 | NP | NP | 6 | Positive | NT | J1 | 3 | 8 | PEN, OXA, CFX, SXT, CHL, ERY, GEN, TE |
| CHL165 | Chile | 1998 | NP | NP | 2 | Positive | V | J4 | ND | 7 | PEN, OXA, CFX, ERY, TET, TE |
| COB120 | Colombia | 1998 | Blood | NP | >256 | Positive | III | W | NT | 0 | PEN, OXA, CFX, SXT, CIP, CLN, ERY, GEN, RIF |
| DEN 106 | Denmark | 1998 | Others | Infection | >256 | Positive | V | N1 | 8 | 7 | PEN, OXA, CFX, CIP, ERY, GEN, TE |
| DEN 108 | Denmark | 1998 | Blood | Colonisation | >256 | Positive | V | C10 | ND | 7 | PEN, OXA, CFX, ERY, TET |
| DEN 114 | Denmark | 1998 | NP | Infection | 48 | Positive | V | C10 | ND | 7 | PEN, OXA, CFX, ERY, TET |
| DEN 141 | Denmark | 1998 | Abces | Colonisation | >256 | Positive | NT | A23 | 4 | 11 | PEN, OXA, CFX, SXT, CIP, ERY, GEN, TE |
| DEN 147 | Denmark | 1998 | Others | Infection | >256 | Positive | NT | A17 | ND | 10 | PEN, OXA, CFX, SXT, CIP, CHL, ERY, GEN, TET, TE |
| DEN 158 | Denmark | 1998 | Wound | Colonisation | >256 | Positive | NT | A22 | ND | 11 | PEN, OXA, CFX, SXT, CIP, ERY, GEN, TET |
| DEN 162 | Denmark | 1998 | Blood | Infection | >256 | Positive | NT | A18 | ND | 11 | PEN, OXA, CFX, SXT, CIP, CHL, TET |
| DEN 174 | Denmark | 1998 | Blood | Infection | >256 | Positive | NT | A20 | ND | 11 | PEN, OXA, CFX, SXT, CIP, ERY, GEN, TET |
| DEN 175 | Denmark | 1998 | Respiratory | Infection | >256 | Positive | NT | A20 | 18 | 11 | PEN, OXA, CFX, ERY, GEN |
| DEN 181 | Denmark | 1998 | Blood | Infection | >256 | Positive | NT | A5 | 4 | 11 | PEN, OXA, CFX, SXT, CIP, CHL, ERY, GEN, TE |
| DEN 199 | Denmark | 1998 | Wound | Colonisation | 0.64 | Positive | NT | A14 | ND | 8 | PEN, OXA, CFX, SXT, CLN, ERY, GEN, TE |
| HUR83 | Hungary | 1998 | Wound | Infection | >256 | Positive | V | F4 | ND | 6 | PEN, OXA, CFX, SXT, CIP, CLN, ERY, TET, TE |
| HUR100 | Hungary | 1998 | Respiratory | Infection | >256 | Positive | NT | A15 | ND | 9 | PEN, OXA, CFX, SXT, CLN, ERY, GEN, TET, TE |
| HUR101 | Hungary | 1998 | Wound | Infection | >256 | Positive | NT | A8 | ND | 11 | PEN, OXA, CFX, SXT, CIP, CHL, GEN |
| HUR133 | Hungary | 1998 | Blood | Infection | >256 | Positive | NT | I1 | 23 | 9 | PEN, OXA, CFX, SXT, CIP, CLN, ERY, GEN, TE |
| HUR136 | Hungary | 1998 | Blood | Infection | >256 | Positive | NT | I3 | 2 | 10 | PEN, OXA, CFX, CLN, ERY, GEN |
| ICE104 | Iceland | 1998 | Urine | Infection | >256 | Positive | NT | C1 | 20 | 6 | PEN, OXA, CFX, SXT, CIP, CHL, CLN, ERY, GEN, TET, TE |
| ICE114 | Iceland | 1998 | Wound | Colonisation | >256 | Positive | NT | A7 | ND | 8 | PEN, OXA, CFX, SXT, CIP, CHL, ERY, TE |
| ICE126 | Iceland | 1998 | Wound | Colonisation | >256 | Positive | NT | A16 | ND | 7 | PEN, OXA, CFX, SXT, CIP, CLN, ERY, GEN, TE |
| ICE129 | Iceland | 1998 | Urine | Colonisation | >256 | Positive | NT | A1 | 4 | 7 | PEN, OXA, CFX, SXT, CIP, ERY, GEN, TET |
| ICE140 | Iceland | 1998 | Urine | Infection | >256 | Positive | NT | C3 | ND | 7 | PEN, OXA, CFX, SXT, CIP, CLN, ERY, GEN, TET, TE |
| ICE144 | Iceland | 1998 | Urine | Colonisation | >256 | Positive | NT | C4 | ND | 8 | PEN, OXA, CFX, SXT, CIP, CHL, CLN, ERY, GEN, TET |
| ICE145 | Iceland | 1998 | Urine | Infection | >256 | Positive | NT | A6 | ND | 11 | PEN, OXA, CFX, SXT, CIP, CHL, ERY |
| ICE152 | Iceland | 1998 | Urine | Infection | 12 | Positive | NT | C5 | 34 | 10 | PEN, OXA, CFX, ERY |
| ICE153 | Iceland | 1998 | Respiratory | Colonisation | 16 | Positive | NT | C6 | 34 | 9 | PEN, OXA, CFX, ERY |
| ICE162 | Iceland | 1998 | Wound | Colonisation | >256 | Positive | NT | C1 | ND | 8 | PEN, OXA, CFX, SXT, CIP, CHL, CLN, ERY, GEN, TET |
| ICE169 | Iceland | 1998 | Wound | Infection | >256 | Positive | NT | A1 | ND | 11 | PEN, OXA, CFX, SXT, CIP, ERY, GEN, TET |
| ICE171 | Iceland | 1998 | Blood | Colonisation | >256 | Positive | NT | A3 | ND | 13 | PEN, OXA, CFX, SXT, CIP, ERY, GEN, TET |
| ICE172 | Iceland | 1998 | Wound | Infection | >256 | Positive | NT | A9 | 24 | 8 | PEN, OXA, CFX, SXT, CIP, CLN, GEN |
| ICE200 | Iceland | 1999 | Blood | NP | >256 | Positive | NT | F5 | 25 | 9 | PEN, OXA, CFX, SXT, GEN, TET, TE |
| ITL397 | Italy | 1998 | Wound | Infection | >256 | Positive | V | T1 | 26 | 8 | PEN, OXA, CFX, CIP, CHL, ERY, GEN, TET |
| ITL398 | Italy | 1998 | Urine | Infection | >256 | Positive | NT | D5 | ND | 8 | PEN, OXA, CFX, CIP, ERY, GEN |
| ITL400 | Italy | 1998 | Abces | Infection | >256 | Positive | NT | Q2 | 2 | 7 | PEN, OXA, CFX, SXT, CIP, ERY, GEN, RIF |
| ITL408 | Italy | 1998 | Blood | Infection | >256 | Positive | NT | F2 | 35 | 8 | PEN, OXA, CFX, CIP, CLN, ERY, GEN |
| ITL420 | Italy | 1998 | Blood | Infection | >256 | Positive | NT | E3 | 27 | 9 | PEN, OXA, CFX, SXT, CIP, CHL, ERY, RIF |
| ITL423 | Italy | 1998 | Respiratory | Infection | >256 | Positive | IV | D1 | 27 | 9 | PEN, OXA, CFX, CIP, ERY, GEN, RIF |
| ITL440 | Italy | 1998 | Wound | Colonisation | >256 | Positive | IV | D1 | ND | 9 | PEN, OXA, CFX, SXT, CIP, CHL, ERY, GEN, RIF |
| ITL441 | Italy | 1998 | Blood | Colonisation | 0.25 | Positive | NT | J5 | 28 | 8 | PEN, OXA, CFX, GEN, TET, TE |
| ITL480 | Italy | 1998 | Respiratory | Colonisation | >256 | Positive | NT | R2 | 19 | 1 | PEN, OXA, CFX, SXT, CIP, CHL, CLN, ERY, GEN |
| ITL565 |  | 1998 | Wound | Infection | >256 | Positive | NT | L2 | NT | 9 | PEN, OXA, CFX, SXT, CIP, CHL, GEN, TET |
| ITL578 | Italy | 1998 | Urine | Colonisation | >256 | Positive | V | D6 | 3 | 10 | PEN, OXA, CFX, SXT, CIP, GEN, TET |
| ITL590 | Italy | 1998 | Respiratory | NP | >256 | Positive | V | D7 | ND | 8 | PEN, OXA, CFX, SXT, CIP, ERY, GEN, TET |
| ITL597 | Italy | 1998 | Abces | NP | >256 | Positive | IV | T2 | 25 | 7 | PEN, OXA, CFX, SXT, CIP, CHL, GEN, TET, TE |
| ITL618 | Italy | 1998 | Urine | NP | 2 | Positive | NT | I4 | 3 | 8 | PEN, OXA, CFX, SXT, CIP, ERY, GEN, TE |
| ITL629 | Italy | 1998 | Blood | Colonisation | >256 | Positive | V | I2 | ND | 9 | PEN, OXA, CFX, SXT, CIP, CLN, ERY, GEN, TE |
| ITL634 | Italy | 1998 | Urine | Colonisation | >256 | Positive | NT | D9 | ND | 7 | PEN, OXA, CFX, CIP, ERY, GEN |
| JAP295 | Japan | 1998 | Abces | Infection | >256 | Positive | NT | Z | 19 | 10 | PEN, OXA, CFX, CIP, GEN |
| MCO167 | Mexico | 1998 | Blood | Infection | 0.75 | Positive | V | M4 | ND | 7 | PEN, OXA, CFX, SXT, CIP, CHL, ERY, GEN, TET |
| MCO185 | Mexico | 1998 | Blood | Infection | >256 | Positive | IV | M1 | 1 | 7 | PEN, OXA, CFX, SXT, CIP, CHL, ERY, GEN |
| MCO188 | Mexico | 1998 | Others | Infection | 0.75 | Positive | V | D4 | ND | 8 | PEN, OXA, CFX, SXT, CIP, CHL, CLN, ERY, GEN |
| MCO189 | Mexico | 1998 | Blood | Infection | >256 | Positive | NT | D10 | ND | 9 | PEN, OXA, CFX, SXT, CIP, CHL, CLN, ERY, GEN |
| MCO190 | Mexico | 1998 | Others | Infection | >256 | Positive | IV | N2 | 8 | 7 | PEN, OXA, CFX, SXT, CLN, ERY, GEN, TET |
| MCO191 | Mexico | 1998 | Blood | Infection | 0.75 | Positive | NT | P2 | 29 | 9 | PEN, CFX, ERY, TET |
| MCO202 | Mexico | 1998 | Blood | Infection | >256 | Positive | IV | M1 | ND | 8 | PEN, OXA, CFX, SXT, CIP, CHL, ERY, GEN |
| PLN129 | Poland | 1998 | Urine | Colonisation | >256 | Positive | NT | L4 | ND | 8 | PEN, OXA, CFX, SXT, CIP, CHL, ERY, GEN |
| PLN130 | Poland | 1998 | Urine | Infection | 1 | Positive | NT | G3 | 36 | 7 | PEN, OXA, CFX, TET |
| PLN132 | Poland | 1998 | Blood | Infection | >256 | Positive | NT | H1 | 19 | 6 | PEN, OXA, CFX, SXT, CIP, CLN, ERY, GEN |
| PLN133 | Poland | 1998 | Blood | Infection | >256 | Positive | NT | H2 | ND | 6 | PEN, OXA, CFX, SXT, CIP, CLN, ERY, GEN |
| PLN134 | Poland | 1998 | Blood | Infection | >256 | Positive | NT | B1 | ND | 8 | PEN, OXA, CFX, CIP, ERY, GEN |
| PLN135 | Poland | 1998 | Blood | Infection | >256 | Positive | NT | H3 | ND | 6 | PEN, OXA, CFX, SXT, CIP, CLN, ERY, GEN, TET |
| PLN136 | Poland | 1998 | Blood | Infection | >256 | Positive | NT | E2 | ND | 5 | PEN, OXA, CFX, SXT, CIP, ERY, GEN |
| PLN137 | Poland | 1998 | NP | Infection | >256 | Positive | NT | A4 | ND | 8 | PEN, OXA, CFX, SXT, CIP, CLN, ERY, GEN, TE |
| PLN138 | Poland | 1998 | Blood | Infection | >256 | Positive | NT | A10 | ND | 10 | PEN, OXA, CFX, SXT, ERY, GEN |
| PLN139 | Poland | 1998 | Blood | Infection | 4 | Positive | NT | A11 | ND | 10 | PEN, OXA, CFX, SXT, ERY, GEN |
| PLN140 | Poland | 1998 | Blood | Infection | >256 | Positive | V | O3 | 2 | 8 | PEN, OXA, CFX, SXT, CIP, CHL, CLN, ERY, GEN, RIF, TE |
| TAW149 | Taiwan | 1998 | Respiratory | Colonisation | >256 | Positive | NT | E1 | 4 | 9 | PEN, OXA, CFX, SXT, CIP, CHL, GEN, RIF |
| TAW160 | Taiwan | 1998 | Respiratory | Colonisation | >256 | Positive | NT | L3 | ND | 9 | PEN, OXA, CFX, SXT, CIP, CHL, CLN, ERY, GEN, RIF |
| URU115 | Uruguay | 1998 | Blood | Infection | >256 | Positive | I | N3 | 8 | 7 | PEN, OXA, CFX, SXT, CIP, ERY, GEN, TET, TE |
| URU161 | Uruguay | 1998 | Blood | Colonisation | 16 | Positive | V | H4 | 30 | 5 | PEN, OXA, CFX, SXT, CHL, GEN |
| HSM726 | Portugal | 2010 | NP | NP | >256 | Positive | NT | B8 | ND | 7 | PEN, OXA, CFX, SXT, CIP, GEN |
| HSM729 | Portugal | 2010 | NP | NP | 0.75 | Positive | V | O1 | 1 | 7 | PEN, CFX, SXT, CIP, ERY, GEN |
| HSM731 | Portugal | 2010 | NP | NP | >256 | Positive | V | Y | 3 | 3 | PEN, OXA, CFX, SXT, CIP, ERY, GEN |
| HSM736 | Portugal | 2010 | NP | NP | >256 | Positive | NT | B8 | ND | 7 | PEN, OXA, CFX, SXT, CIP, ERY, GEN |
| HSM742 | Portugal | 2010 | NP | NP | >256 | Positive | V | C7 | 1 | 9 | PEN, OXA, CFX, SXT, CIP, ERY, GEN |
| HSM744 | Portugal | 2010 | NP | NP | >256 | Positive | NT | B13 | ND | 7 | PEN, OXA, CFX, SXT, CIP, ERY, GEN |
| HSM745 | Portugal | 2010 | NP | NP | >256 | Positive | V | A12 | 29 | 9 | PEN, OXA, CFX, SXT, ERY |
| HSM746 | Portugal | 2010 | NP | NP | >256 | Positive | NT | B8 | ND | 7 | PEN, OXA, CFX, CIP, GEN |
| HSM747 | Portugal | 2010 | NP | NP | >256 | Positive | V | C7 | ND | 8 | PEN, OXA, CFX, SXT, CIP, ERY, GEN |
| HSM748 | Portugal | 2010 | NP | NP | >256 | Positive | NT | B8 | ND | 7 | PEN, OXA, CFX, CIP, GEN |
| HSM752 | Portugal | 2010 | NP | NP | 12 | Positive | V | A12 | ND | 9 | PEN, OXA, CFX, SXT, ERY |
| HSM757 | Portugal | 2010 | NP | NP | >256 | Positive | NT | B3 | ND | 7 | PEN, OXA, CFX, CIP, CLN, ERY, GEN |
| HSM783 | Portugal | 2010 | NP | NP | >256 | Positive | NT | B5 | ND | 7 | PEN, OXA, CFX, SXT, CIP, ERY, GEN |
| HSM790 | Portugal | 2010 | NP | NP | >256 | Positive | NT | B8 | ND | 7 | PEN, OXA, CFX, SXT, CIP, GEN |
| HSM803 | Portugal | 2010 | NP | NP | >256 | Positive | NT | O2 | 1 | 7 | PEN, OXA, CFX, SXT, CIP, ERY, GEN |
| HSM810 | Portugal | 2010 | NP | NP | >256 | Positive | V | C9 | ND | 7 | PEN, OXA, CFX, SXT, CIP, ERY |
| HSM815 | Portugal | 2010 | NP | NP | >256 | Positive | NT | B1 | 3 | 7 | PEN, OXA, CFX, SXT, CIP, CLN, ERY, GEN |
| HSM820 | Portugal | 2010 | NP | NP | >256 | Positive | NT | B6 | ND | 7 | PEN, OXA, CFX, SXT, CIP, ERY, GEN |
| HSM821 | Portugal | 2010 | NP | NP | >256 | Positive | NT | V1 | 18 | 7 | PEN, OXA, CFX, SXT, CIP, CLN, ERY, GEN, TET |
| HSM824 | Portugal | 2010 | NP | NP | >256 | Positive | NT | B3 | ND | 7 | PEN, OXA, CFX, CIP, ERY, GEN, TET |
| HSM827 | Portugal | 2010 | NP | NP | >256 | Positive | NT | B7 | ND | 7 | PEN, OXA, CFX, SXT, CIP, ERY, GEN |
| HSM832 | Portugal | 2010 | NP | NP | >256 | Positive | NT | E5 | ND | 7 | PEN, OXA, CFX, CIP, GEN |
| HSM838 | Portugal | 2010 | NP | NP | >256 | Positive | NT | A19 | ND | 8 | PEN, OXA, CFX, SXT, CIP, CLN, ERY, GEN, RIF |
| HSM842 | Portugal | 2010 | NP | NP | >256 | Positive | NT | E3 | 3 | 7 | PEN, OXA, CFX, SXT, CIP, ERY, GEN |
| HSM843 | Portugal | 2010 | NP | NP | >256 | Positive | NT | E3 | ND | 7 | PEN, OXA, CFX, SXT, CIP, ERY, GEN, TET |
| HSM846 | Portugal | 2010 | NP | NP | >256 | Positive | NT | E5 | ND | 7 | PEN, OXA, CFX, SXT, CIP, ERY, GEN |
| HSM847 | Portugal | 2010 | NP | NP | >256 | Positive | NT | V2 | ND | 7 | PEN, OXA, CFX, SXT, CIP, CLN, ERY, GEN, TET |
| HSM852 | Portugal | 2010 | NP | NP | >256 | Positive | NT | E5 | ND | 7 | PEN, OXA, CFX, SXT, CIP, ERY, GEN |
| CHI53 | China | 1998 | Urine | Infection | 64 | Negative | NT | a1* | 19 | 4 | PEN, OXA, CFX, SXT, CLN, ERY, GEN, TET |
| CHI78 | China | 1998 | Urine | Infection | 12 | Negative | NT | a3* | 19 | 6 | PEN, OXA, CFX, SXT, CIP, CLN, ERY, GEN |
| CHI79 | China | 1998 | Blood | Infection | 64 | Negative | NT | a2* | 19 | 5 | PEN, OXA, CFX, SXT, CIP, CLN, ERY, GEN |
| ITL503 | Italy | 1998 | Respiratory | Colonisation | 0.19 | Negative | NT | b1* | 37 | 6 | PEN, CLN |
| ITL566 | Italy | 1998 | Blood | Infection | 0.125 | Negative | NT | b2* | 19 | 9 | PEN, OXA, CHL |
| ITL573 | Italy | 1998 | Others | Infection | 0.125 | Negative | NT | c* | 8 | 8 | PEN, OXA, SXT, CIP, CLN, ERY, GEN |

NP, not provided; NT, non-typeable; ST, insertion sequence; ND, not determined; PEN, penicillin; OXA, oxacillin; CFX, cefoxitin; SXT, trimethoprim- sulfamethoxazole; CIP, ciprofloxacin; CHL, chloramphenicol; CLN, clindamycin; ERY, erythromycin; GEN, gentamicin; RIF, rifampin; TET, tetracycline; TE, teicoplanin.

*PFGE analysis of MRSHae and MSSHae was done idependently and classification should not be compared between them.
